# Near‐Infrared Upconversion Luminescence and Bioimaging In Vivo Based on Quantum Dots

**DOI:** 10.1002/advs.201801834

**Published:** 2019-01-18

**Authors:** Xiaochen Qiu, Xingjun Zhu, Xianlong Su, Ming Xu, Wei Yuan, Qingyun Liu, Meng Xue, Yawei Liu, Wei Feng, Fuyou Li

**Affiliations:** ^1^ Department of Chemistry and State Key Laboratory of Molecular Engineering of Polymers Fudan University 220 Handan Road Shanghai 200433 P.R. China

**Keywords:** bioimaging, low power, nanoparticles, quantum dots, upconversion luminescence

## Abstract

Recently, upconversion luminescence (UCL) has been widely applied in bioimaging due to its low autofluorescence and high contrast. However, a relatively high power density is still needed in conventional UCL bioimaging. In the present study, an ultralow power density light, as low as 0.06 mW cm^−2^, is applied as an excitation source for UCL bioimaging with PbS/CdS/ZnS quantum dots (UCL‐QDs) as probes. The speculated UCL mechanism is a phonon‐assisted single‐photon process, and the relative quantum yield is up to 4.6%. As determined by continuous irradiation with a 980 nm laser, the UCL‐QDs show excellent photostability. Furthermore, UCL‐QDs‐based probe is applied in tumor, blood vessel, and lymph node bioimaging excited with an eye‐safe low‐power light‐emitting diode light in a nude mouse with few heat effects.

## 1 Introduction

Upconversion luminescence (UCL) can convert low‐energy photons, usually in the near‐infrared (NIR) region, into higher ones.^1^, ^2^ In the past decade, UCL has gained considerable attention and has been widely applied in bioimaging,^3^, ^4^, ^5^, ^6^ biodetection,^7^, ^8^ molecular diagnostics,^9^ and clinical therapeutics.^10^, ^11^, ^12^ In bioimaging, UCL shows great promise owing to its various merits such as low autofluorescence, deep tissue penetration, and little photodamage to living organisms. During the past decades, rare earth–doped nanoparticles and triplet–triplet annihilation‐based upconversion systems have been widely examined by many research groups for bioapplication.^13^ However, for rare earth–doped nanoparticles, due to the narrow band absorption cross section of the Yb^3+^ sensitizer^14^ and low UCL quantum yield,^15^ a relatively high power density excitation is still necessary, to ensure that a strong enough emission signal can be achieved for bioapplication. Triplet–triplet annihilation‐based UCL has a low power density excitation^16^; however, an organic solution system is required,^17^, ^18^, ^19^ and in aqueous solution, it is still challenging to fabricate a low power density light excited small‐size nanoprobe,^20^ which has limited its bioapplication in vivo.

Colloidal quantum dots (QDs) have attracted considerable attention from researchers and have been developed sharply over the past decades, due to their unique merits, such as broad excitation, size‐dependent photoluminescence, narrow and symmetric emission peaks, and high luminescence quantum yield.^21^ However, most research and applications have focused on the Stokes shift emission,^22^, ^23^ and a little attention has been paid to the UCL of QDs,^24^, ^25^ which is necessary to fabricate a core/shell structure owing to the relying on an auger recombination process. Differently, in the present study, we developed an UCL imaging agent based on PbS QDs (PbS, PbS/CdS or PbS/CdS/ZnS QDs), which has a broad excitation region in the NIR region and an ultralow excitation threshold, and was used for low‐power light‐emitting diode (LED) light‐excited UCL bioimaging in a small animal with few heat effects. Although the core/shell structure is not necessary in this UCL process, a core/shell/shell structure of PbS/CdS/ZnS QDs (UCL‐QDs) was fabricated in order to improve light stability and reduce biotoxicity. The power‐ and temperature‐dependent UCL spectra suggest that UCL emission may be achieved via a phonon‐assisted single‐photon process. It should be noted that the NIR emission of UCL‐QDs has a relative quantum yield of 4.6%. Using a self‐assembly method, the surface of the UCL‐QDs was coated with polyethylene glycol (PEG), which greatly improved the water solubility and biocompatibility of these nanostructures for stepwise bioimaging. Importantly, these UCL‐QDs were successfully used for UCL tumor, blood vessel, and lymph node bioimaging in vivo excited by a low‐power eye‐safe LED light at 980 nm.

## 2 Results and Discussion

### 2.1 Synthesis and Characterization of UCL‐QDs

**Figure 1:**
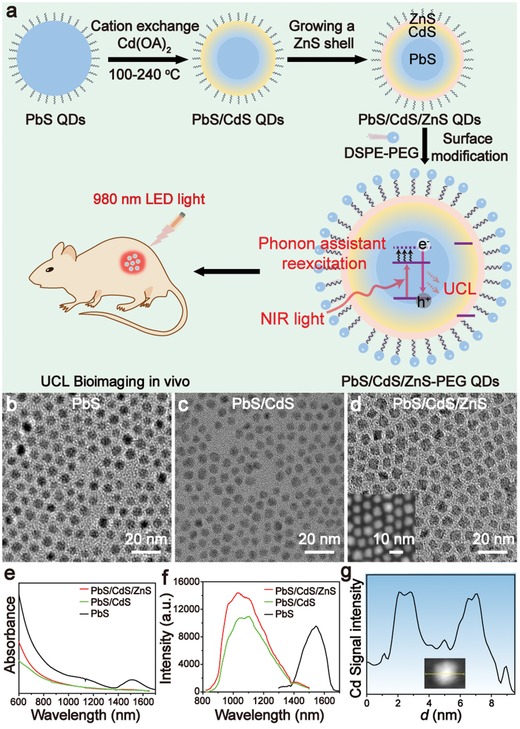
a) Schematic diagram of the synthesis of UCL‐QDs and phonon‐assisted single‐photon UCL bioimaging. TEM images of b) PbS, c) PbS/CdS, and d) UCL‐QDs, and the inset of (d) shows the high‐angle annular dark field (HAADF) TEM image of UCL‐QDs. e) Absorption and f) fluorescence spectra of PbS, PbS/CdS, and UCL‐QDs. g) EDX line scan of a randomly selected UCL‐QD. Inset: STEM image of the nanoparticle under investigation.

UCL‐QDs were synthesized using a modified previously published three‐step method (**Figure** **1**a; see details in the Supporting Information).^26^, ^27^, ^28^ First, the PbS core QDs were presynthesized by injecting the sulfur precursor solution into the hot lead precursor solution at a typical temperature and purification via centrifugation. The PbS/CdS QDs were then prepared via cation exchange at a stepwise elevated temperature. The UCL‐QDs were finally obtained after growing a thin ZnS shell on the PbS/CdS QDs. Transmission electron microscopy (TEM) measurements reveal the uniform sizes of PbS core QDs (5.5 ± 0.5 nm), PbS/CdS QDs (5.5 ± 0.6 nm), and UCL‐QDs (6.1 ± 0.6 nm) (Figure 1b,c,d). The PbS cores have an absorption peak at around 1515 nm, and a blue‐shifted absorption spectrum have been obtained after cation exchange with Cd^2+^ (Figure 1e). Corresponding to this, the emission peak also changed from 1548 to 1075 nm excited by a 680 nm laser (Figure 1f). According to the experimental formula reported in the literature,^29^ the calculated radius of the PbS core in the PbS/CdS structure is approximately 3.1 nm and the thickness of the CdS shell is around 1.2 nm (≈4 monolayers). The thick CdS shell is necessary to improve the thermal stability of the QDs for further overcoating of the ZnS shell at an elevated temperature over 200 °C. After growing a 0.3 nm ZnS shell on the PbS/CdS QDs, the emission peak shows a little blue‐shift (≈45 nm) and the intensity has been improved by approximately 1.4 times. The energy‐dispersive X‐ray (EDX) analysis line scan (Figure 1g) and elemental analysis (Figure S1, Supporting Information) of a randomly selected UCL‐QD further confirm the core/shell/shell structure. The distribution of Cd within a typical UCL‐QD obtained from the EDX line scan demonstrates a ≈1.3 nm Cd shell in the UCL‐QD, which matches the results calculated from TEM measurements. The rock‐salt cubic crystalline structure of the PbS core and zinc blende crystal structure of the CdS shell are confirmed by X‐ray diffraction (XRD) patterns (Figure S2a, Supporting Information) and high‐resolution TEM images (Figure S2b,c, Supporting Information).

For further bioapplication, the UCL‐QDs were further modified with 1,2‐distearoyl‐sn‐glycero‐3‐phosphoethanolamine‐*N*‐[methoxy(polyethylene glycol)‐2000] (DSPE‐PEG‐2000) through a hydrophobic interaction. After modification with PEG, the hydrophilic UCL‐QDs could be dispersed in deionized water for several weeks with no obvious aggregation, which was confirmed by dynamic light scattering (DLS) measurements (Figure S3, Supporting Information). To further confirm the successful modification of UCL‐QDs by PEG, Fourier‐transform infrared (FT‐IR) spectrometry was performed. As shown in the FT‐IR spectrum (Figure S4, Supporting Information), PEG‐UCL‐QDs show strong absorption bands of asymmetric and symmetric —CH_2_— stretching bands at 2918 and 2850 cm^−1^, and the bands around 1000–1500 cm^−1^ are assigned to the —C—O—C group vibrations. These results indicate the successful conjugation of PEG to the surface of the UCL‐QDs.

### 2.2 UCL Properties

**Figure 2:**
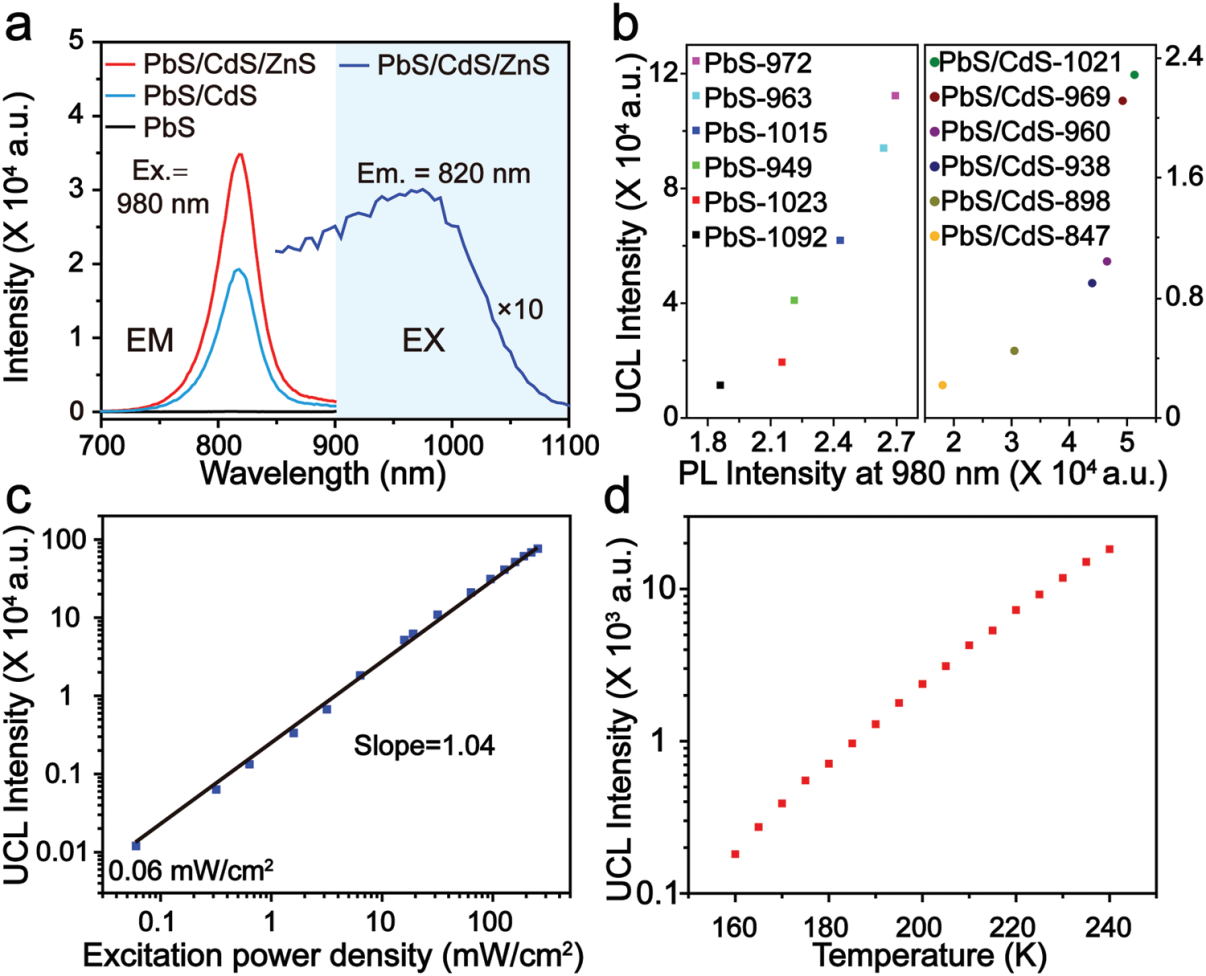
a) UCL spectra of PbS, PbS/CdS, and UCL‐QDs excited by a 980 nm laser and the UCL excitation spectrum of UCL‐QDs. b) UCL intensity versus PL intensity at 980 nm of PbS based QDs with different emissions. UCL intensity versus c) excitation power density and d) temperature of PbS‐977.

The Stokes‐shift NIR luminescence property of PbS‐based QDs has been widely investigated due to their lower photon scattering and increased imaging depth. However, the NIR light–excited UCL of PbS‐based QDs has attracted little attention. Here, the UCL properties of PbS‐based QDs were investigated. In order to increase the photoluminescence intensity and enhance photostability, the core/shell/shell structure was synthesized. Although PbS‐1548 showed almost no UCL emission under the excitation of a 980 nm laser, obvious UCL emission at 820 nm was detected after the emission blue‐shift to 1026 nm via the cation exchange process. The intensity of UCL emission increased 1.8 times, after growing a monolayer of ZnS shell due to a reduction in surface quenching (**Figure** **2**a). In addition, the excitation spectrum of UCL‐QDs reveals that they can be excited at a broad region from 850 to near 1050 nm (Figure 2a). The ZnS shell is essential for maintaining the UCL emission and enhancing photostability. Under 980 nm (200 mW cm^−2^) laser exposure for 60 min, the UCL emission of UCL‐QDs is 96% with a little decrease, whereas that of PbS/CdS decreases to 85% (Figure S5, Supporting Information). To further investigate the UCL emission of PbS‐based QDs, PbS and PbS/CdS QDs with different emissions were synthesized and the Stokes and anti‐Stokes emission were detected (Figure S6, Supporting Information). The results demonstrate that the UCL peak shifts a little and is focused at around 820 nm, although the Stokes shift emission shows a large red‐shift from 849 to 1010 nm. It should also be mentioned that the UCL intensity of PbS‐based QDs has a positive correlation with the Stokes shift emission intensity at the UCL excitation area (Figure 2b).

To reveal the UCL mechanism of UCL‐QDs, the dependence of UCL intensity on the excitation power intensity, which can provide information on the UCL mechanism, is necessary to be investigated. For this, the power intensity–dependent UCL was investigated. The results show a linear relationship between UCL intensity and excitation power, and the UCL‐QDs can be excited at a power density of 0.06 mW cm^−2^ (Figure 2c). According to a previous study,^30^ for UCL obtained through a two‐photon absorption process, a quadratic dependence *I*_UCL_–*I*_Ex_^2^ should be observed, and if induced by the Auger recombination process, a cubical dependence *I*_UCL_–*I*_Ex_^3^ is observed. Both of these mechanisms require a relatively high excitation power intensity. In contrast to this, only the phonon‐assisted UCL can be excited at a very low power density and the UCL intensity increases linearly with excitation power via a single‐photon process. Thus, we speculate that the UCL emission of UCL‐QDs is via a phonon‐assisted single‐photon process. For further confirmation, the temperature‐dependent UCL spectra were detected from 160 to 240 K. The spectra show a growing UCL intensity with increasing temperature (Figure 2d), due to a rise in the number of phonons. Although the detailed mechanism is currently still unclear, the above results show that UCL formation of UCL‐QDs is thought to be a phonon‐assisted single‐photon process. The possible mechanism is shown in Figure S7 in the Supporting Information. Briefly, the electron is first excited from the valence band to the conduction band, after the absorption of NIR light. The electron is then further excited to the defect state with the assistance of phonons or heat. UCL finally occurs when this exciton recombines. With a NIR dye (NRh‐1) as a reference,^31^ the upconversion luminescence quantum yield (Φ_UCL_) of the UCL‐QDs in pure water was measured to be 4.6% (100 mW cm^−2^). It is worth mentioning that the Φ_UCL_ of the UCL‐QDs is independent of the excitation power density, which is different to the rare earth–doped upconversion nanoparticles, whose Φ_UCL_ increases with increasing excitation power density. Therefore, the UCL‐QDs have the advantages of being excited under a low power density light.

### 2.3 UCL Imaging In Vivo

**Figure 3:**
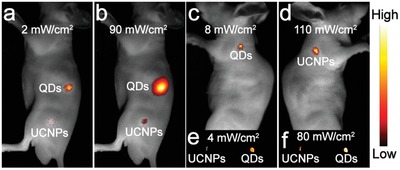
Comparative UCL imaging of UCL‐QDs and Tm‐UCNPs under 980 nm laser irradiation with different power density. a,b) UCL imaging after subcutaneous injection with 30 µL of UCL‐QDs or Tm‐UCNPs aqueous solution. Lymph node UCL imaging c,d) in vivo and e,f) ex vivo.

To evaluate the excitation threshold of UCL‐QDs and rare earth–doped upconversion nanoparticles (UCNPs), β‐NaYbF_4_:0.5%Tm@NaYF_4_ UCNPs (Tm‐UCNPs) with a size about 24 nm (Figure S8, Supporting Information) were synthesized via a solvothermal method according to the literature.^32^ Then, we performed UCL imaging under 980 nm laser irradiation at different power densities. 30 µL of PEG‐Tm‐UCNPs or PEG‐UCL‐QDs aqueous solution (both at a concentration of 3 mg mL^−1^) was subcutaneously injected into a nude mouse. **Figure** **3**a shows that the UCL‐QDs still give an obvious UCL signal with a power density of 2 mW cm^−2^. However, no UCL signal can be detected at the Tm‐UCNPs injection area and the UCL signal of Tm‐UCNPs starts to become obvious when the power intensity increases to 90 mW cm^−2^ (Figure 3b). To further compare the excitation threshold, we used UCL‐QDs and Tm‐UCNPs for lymphatic UCL imaging. The excitation threshold for UCL‐QDs is 8 mW cm^−2^ for in vivo lymphatic imaging, much lower than that for Tm‐UCNPs, which is up to 110 mW cm^−2^. In ex vivo UCL lymphatic imaging, the UCL‐QDs can be excited at 4 mW cm^−2^ light, while a power intensity of 80 mW cm^−2^ is still needed for Tm‐UCNPs. These results show that UCL‐QDs can be excited at an ultralow power density, which is markedly superior to Tm‐UCNPs.

**Figure 4:**
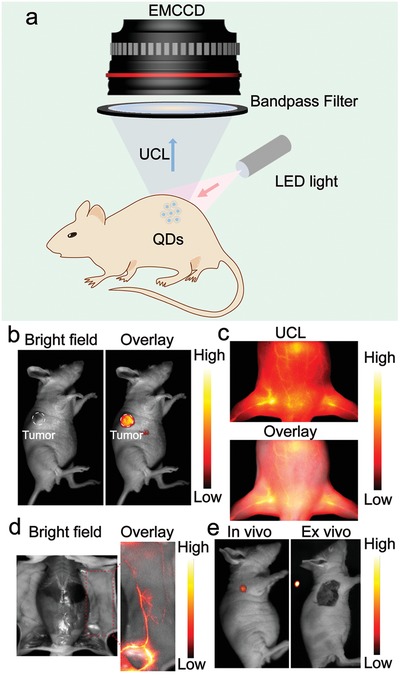
a) Schematic diagram of low‐power LED light excited bioimaging in vivo. A 980 nm LED light (20 mW cm^−2^) excited b) tumor, c) blood vessel in vivo, d) blood vessel in situ, and e) lymph node UCL imaging of UCL‐QDs.

Low power density is essential for optical imaging in vivo, which can reduce the heat effect and tissue damage. Compared with rare earth–doped UCNPs, UCL‐QDs can be excited at a much lower power density, and a low‐power LED light, which is eye‐safe for surgery in the clinic, is enough for excitation. We then performed tumor, blood vessel, and lymph node UCL imaging with an eye‐safe 980 nm LED light (20 mW cm^−2^; **Figure** **4**a). The bioimaging results are shown in Figure 4. UCL imaging of the tumor‐grafted nude mouse was obtained 6 h after intravenous injection of PEG‐UCL‐QDs. A significant UCL signal was collected from the tumor area (Figure 4b). The results show that the UCL‐QDs can be passively targeted to the tumor due to the enhanced permeability and retention (EPR) effect.^33^ The ex vivo biodistribution of UCL‐QDs was assessed by imaging the UCL signals of main organs. Most UCL signals are found in the liver and tumor (Figure S9, Supporting Information), which is in good agreement with the UCL images in vivo. LED light–excited vascular and lymph node UCL imaging was then performed. Vascular imaging was performed immediately after intravenous injection of PEG‐UCL‐QDs aqueous solution. The blood vessels are clearly seen in UCL imaging in vivo (Figure 4c) or in situ (Figure 4d). Lymphatic UCL imaging can assist in the location of lymph nodes and facilitate lymph node dissection (Figure 4f). These results show that UCL‐QDs can potentially be used as an UCL imaging agent excited by a low‐power LED light.

### 2.4 Heating Effect Evaluation

**Figure 5:**
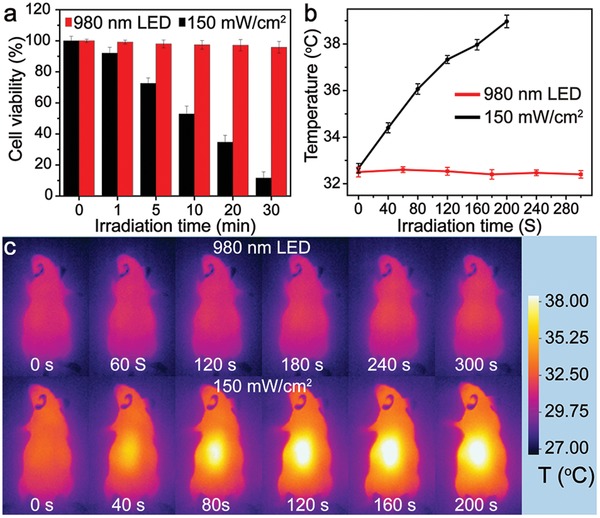
Evaluation of the heating effect of 980 nm LED light (20 mW cm^−2^) and a 980 nm laser (150 mW cm^−2^). a) Viability of HeLa cells, b) apparent temperature of nude mouse skin, and c) infrared thermal image of nude mouse irradiation with 980 nm LED light and the 980 nm laser. Average values of temperature under different time points were given based on three times measurements. Error bars were defined as SD.

The in vitro heating effect induced by a 980 nm laser (150 mW cm^−2^) and 980 nm LED light (20 mW cm^−2^) on HeLa cells was evaluated using 3‐(4,5‐dimethylthiazol‐2‐yl)‐2,5‐diphenyltetrazolium bromide (MTT) assay. In the MTT assays, HeLa cells were irradiated with a 980 nm laser or LED light for different time periods from 0 to 30 min. When the irradiation time is increased to 5 min, the 980 nm laser has a significant killing effect. However, with LED light, most of the cells survive even after being irradiated for 30 min (**Figure** **5**a). The in vivo heating effect was then studied by irradiating a nude mouse with a 980 nm laser or LED light. The skin temperature of the nude mouse was monitored by an infrared thermal imager. Following irradiation by the 980 nm laser for 200 s, a notable heating effect is observed and the skin temperature increases by 8 °C (Figure 5b,c), which is high enough to cause a burn wound to the nude mouse. In contrast, no significant heating effect is observed with the 980 nm LED light, even after irradiation for 5 min and only a slight rise in skin temperature in the nude mouse is detected (Figure 5b,c). These results confirm that low power density eye‐safe 980 nm LED light shows only a slight heating effect, which is essential for UCL imaging in vivo.

### 2.5 Toxicity of UCL‐QDs

**Figure 6:**
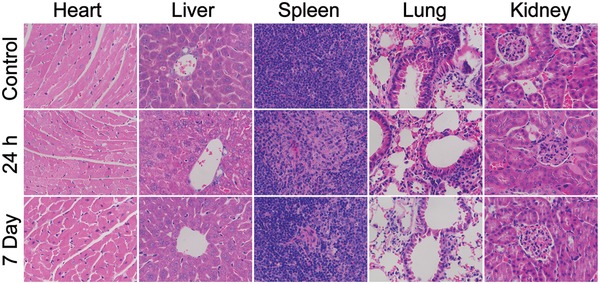
H&E‐stained tissue sections of the heart, liver, spleen, lung, and kidney harvested from mice after intravenous injection without (control) or with PEG‐UCL‐QDs (3 mg mL^−1^, 0.2 mL) after 24 h or 7 days.

For potential bioapplication, the evaluation of cytotoxicity was performed on HeLa cells using the MTT assay. Briefly, HeLa cells were incubated with PEG‐UCL‐QDs at different concentrations varying from 0 to 400 µg mL^−1^ for 24 h. No significant cytotoxicity is observed, and cell viability is over 90% even with a concentration of 400 µg mL^−1^ (Figure S10, Supporting Information). We also performed histological analysis of nude mouse tissues using hematoxylin and eosin (H&E) staining. The results showed no notable histological changes in the harvested organs (**Figure** **6**) following an intravenous injection of 200 µL (3 mg mL^−1^ PEG‐UCL‐QDs) aqueous solution. These results suggest that PEG‐UCL‐QDs exhibit low toxicity in living systems.

## 3 Conclusion

In conclusion, we fabricated an ultralow power density 980 nm light excited UCL based on UCL‐QDs, which can even be excited by a power density (0.06 mW cm^−2^) lower than sunlight. Our results demonstrate that UCL emission may be achieved through a phonon‐assisted single‐photon process. It should be noted that PEG‐modified UCL‐QDs show little biotoxicity and excellent biocompatibility. Importantly, the quantum yield of photostable UCL‐QDs is approximately 4.6%, and is unrelated to the excitation power density. Moreover, tumor, blood vessel, and lymph node UCL imaging in nude mouse was obtained following excitation with a low power density eye‐safe 980 nm LED light (≈20 mW cm^−12^), which has only a slight heating effect. We believe that the development of these UCL‐QDs will open a new door for UCL bioimaging in the future.

## 4 Experimental Section

*Materials and Characterization*: Lead(II) oxide, cadmium oxide, zinc oxide, and sulfur were obtained from Shanghai Macklin Biochemical Co., Ltd. Octadecene (ODE; technical grade, 90%), oleic acid (OA; technical grade, 90%), and bis(trimethylsilyl)sulfide ((TMS)_2_S) (synthesis grade) were purchased from Sigma Aldrich. Ethanol anhydrous (99.5%) was bought from Adamas‐beta. Chloroform, toluene, and acetone were brought from Sinopharm Chemical Reagent Co., China. DSPE‐PEG‐2000 was obtained from Shanghai Ponsure Biological Technology Co., Ltd. All chemicals were used without further purification.

The size and morphologies of nanoparticles were characterized with a TEM (JEOL, JEM‐2010F) at a working voltage of 200 kV. Energy‐dispersive spectrometer elemental mapping was also performed at TEM (JEOL, JEM‐2010F). Samples were prepared by placing a drop of dilute dispersion in toluene on the surface of a copper grid. Powder XRD measurements were carried out in a PANalytical X'pert PRO X‐ray diffractometer (Cu Kα radiation, λ = 0.15406 nm). Emission spectra of QDs were measured with an Edinburgh LFS‐920 fluorescence spectrometer, UCL using an external 0–9 W adjustable CW laser at 980 nm (Connet Fiber Optics, China) as the excitation source, instead of the Xenon source in the spectrophotometer. FT‐IR spectroscopy was performed using an IR Prestige‐21 spectrometer (Shimadzu) from samples in KBr pellets. DLS measurements were detected by Malvern Zetasizer Nano ZS90. UV−vis absorption spectra were recorded on a Perkin‐Elmer Lambda750 spectrophotometer. The UCL imaging was taken on a small animal UCL in vivo imaging system built by this study group.

*Synthesis of PbS QDs*: The synthesis of PbS QDs was according to the modified Hines' method.^1^ In a typical synthesis, the lead precursor solution was prepared as follows: 0.446 g of lead oxide was added to a mixture of 1.5 mL of OA and 20 mL of ODE. And then the solution was heated to 120 °C and degassed in vacuum until the solution became transparent. Then the solution was heated to the injection temperature of 180 °C under N_2_. Simultaneously, the sulfur precursor solution was prepared via dissolving 200 µL of TMS in 5 mL of ODE, and then the solution was degassed under vacuum with thorough stirring for 1 h at room temperature, and then the solution was switched to N_2_ atmosphere. After the temperature of the lead precursor was stabilized at 180 °C for 10 min, the sulfur precursor solution was rapidly injected into the lead oleate solution under vigorous stirring. After 1 min, the reaction mixture was naturally cooled down to room temperature. The synthesized QDs were washed three times by sequential precipitation with acetone and redispersed in toluene. And the QDs were dispersed in 4 mL of toluene and stored at 4 °C.

*Synthesis of PbS/CdS Core/Shell QDs*: PbS/CdS core/shell QDs had been synthesized according to the previously reported cation exchange method in the literature with slight modification. For preparing cadmium oleate solution, 770.5 mg of CdO, 9 mL of OA, and 10 mL ODE were added to a 100 mL flask. The mixture was heated up to 250 °C under N_2_ for 30 min to obtain a transparent solution and then cooled down to room temperature. 10 mL ODE solution of PbS QDs (200 nmol) was added into the reaction. Then, the solution was degassed under vacuum for 30 min to remove the residual toluene and water. And then the solution was heated to 100 °C under N_2_ for 3 h. After the formation of a thin CdS layer, the solution was heated to 150 °C for another 1 h, and then was further increased to 200 °C maintaining for 30 min. At last, the reaction was raised to 250 °C for 20 min. After cooling down to room temperature, the PbS/CdS core/shell QDs were precipitated and purified by centrifugation via adding 4 mL of toluene and 24 mL of acetone. After washing with acetone for three times, PbS/CdS core/shell QDs were finally dispersed in 1 mL toluene.

*Synthesis of PbS/CdS/ZnS Core/Shell/Shell QDs*

*Preparation of Zinc Precursor*: For preparing zinc precursor solution, 529 mg of ZnO was dissolved in a mixture of 9.5 mL OA and 5.5 mL of ODE under the protection of N_2_ at 240 °C. Then, the solution was cooled down to 100 °C and degassed under vacuum for 30 min. After that, the zinc oleate solution was kept at 120 °C for the further synthesis of PbS/CdS/ZnS core/shell/shell QDs.

*Preparation of Sulfur Precursor*: The sulfur precursor solution was synthesized as follows: 240 mg of sulfur was dissolved in 15 mL of ODE at 150 °C for 30 min under nitrogen atmosphere. After the solution turned to red, it was cooled down to 100 °C and kept at this temperature for the further synthesis of PbS/CdS/ZnS core/shell/shell QDs.

*Overcoating of ZnS Shell*: 100 nmol of PbS/CdS QDs were dispersed in 20 mL of ODE in a 50 mL three‐neck round‐bottom flask. And the solution was degassed at 100 °C for 20 min. Then, the temperature was increased to 240 °C under the protection of N_2_. Then 250 µL of zinc precursor solution was injected into the reaction. After 5 min, 250 µL of sulfur precursor solution was added and the temperature was maintained for 5 min. Then, the sequential injection of zinc and sulfur precursors was repeated two times. Finally, after the solution was cooled down to room temperature, the QDs were precipitated and purified by centrifugation through adding 5 mL of toluene and 35 mL of acetone. And the precipitates were washed with ethanol for two times. Then, the final synthesized PbS/CdS/ZnS core/shell/shell QDs were dispersed in toluene and stored at 4 °C.

*Surface Modification of PbS/CdS/ZnS Core/Shell/Shell QDs*: The synthesis of PbS/CdS/ZnS‐PEG QDs (PEG‐UCL‐QDs) was according to the modified self‐assemble method reported in previous literatures. Typically, 1 mg of as‐synthesized PbS/CdS/ZnS core/shell/shell QDs and 2 mg of DSPE‐PEG‐2000 were dispersed in 15 mL of chloroform. Then, the solution was sonicated for 20 min until the PbS/CdS/ZnS core/shell/shell QDs assembled with DSPE‐PEG‐2000 thoroughly. And the organic solvent was removed via vacuum rotary evaporation at 35 °C. The final products were redispersed in water and stored at 4 °C.

*Measurement of UCL Quantum Yield*: The UCL quantum yield (φ_UCL_) of UCL‐QDs was calculated relative to NRh‐1, which demonstrates an UCL emission at 730 nm excited with an 808 nm laser.^31^ The UCL quantum yield of the UCL‐QDs were calculated using the following equation:

(1) $$\phi_{\mathrm{QD}}=\phi_{\mathrm{Ref}}\times \frac{\mathrm{UCL}(\mathrm{QD})}{\mathrm{UCL}(\mathrm{Ref})}\times \frac{\mathrm{Absorbance}\left(\mathrm{Ref}\right)}{\mathrm{Absorbance}\left(\mathrm{QD}\right)}\times \frac{n_{\mathrm{QD}}{}^{2}}{n_{\mathrm{Ref}}{}^{2}}$$

where φ_QD_ is the UCL QY of the UCL‐QD, φ_Ref_ is the UCL QY of NRh‐1, Absorbance is the absorbance at the excitation wavelength (the QDs were excited with 850 nm laser and NRh‐1 was excited with 808 nm laser at the same power density), and *n* is the refractive index of the solvent. UCL(QD) and UCL(Ref) stand for integrated UCL intensity of the UCL‐QDs and NRh‐1, respectively.

*Cell Culture and Cytotoxicity of PEG‐UCL‐QDs*: A human cervical carcinoma cell line (HeLa cells) was obtained from the Institute of Biochemistry and Cell Biology, Shanghai Institutes for Biological Sciences (SIBS), Chinese Academy of Sciences (CAS, China). The HeLa cells were grown in Modified Eagle's Medium (MEM) supplemented with 10% fetal bovine serum (FBS) at 37 °C and 5% CO_2_. In vitro cytotoxicity was measured by performing MTT assay on HeLa cells. Briefly, cells were seeded in 96‐well plates (5 × 10^4^ cells/well) and allowed to grow at 37 °C and 5% CO_2_ for 1 day. Then, the cells were treated with PEG‐UCL‐QDs at different concentrations (0, 50, 100, 200, and 400 µg mL^−1^, diluted in RPMI 1640) in dark. After 1 day of further incubation, MTT (20 µL, 5 mg mL^−1^) was added to each well and the cells were subsequently kept at 37 °C under 5% CO_2_ for another 4 h. The produced purple formazan product was dissolved in dimethyl sulfoxide and the absorbance at 570 nm was measured using a Tecan Infinite M200 monochromator‐based multifunction microplate reader. The following formula was used to calculate the inhibition of cell growth: Cell viability (%) = (mean of Abs. value of treatment group/mean Abs. value of control) × 100%.

*Histological Analysis*: In the test group, nude mice (*n* = 3) were intravenously injected with PEG‐UCL‐QDs at a total dose of 3 mg mL^−1^ (200 µL). And nude mice (*n* = 3) with no injection were selected as the control group. Tissues were harvested from test and control groups after 24 h or 1 week. The heart, liver, spleen, lung, and kidney were removed, and fixed in paraformaldehyde, embedded in paraffin, sectioned, and stained with hematoxylin and eosin.

*Comparative UCL Imaging*: In vivo and ex vivo UCL imaging was taken with the excitation at 980 nm laser at different power density. Subcutaneous UCL imaging was taken after subcutaneous injection with 30 µL of UCL‐QDs or Tm‐UCNPs aqueous solution (3 mg mL^−1^). In vivo lymphatic UCL imaging was performed 30 min after intradermal injection of UCL‐QDs (50 µL, 3 mg mL^−1^) or Tm‐UCNP (50 µL, 3 mg mL^−1^) into the paw of the nude mouse. The lymphatic drainage ex vivo UCL imaging was also measured after removal of the lymph node from the body. The power density on the surface of the nude mouse was measured with a portable densitometer. Images of luminescent signals were analyzed with Kodak Molecular Imaging Software.

*LED Light–Excited Tumor, Blood Vessels, and Lymph Node UCL Imaging*: Animal procedures were in accordance to the guidelines of the Institutional Animal Care and Use Committee (IACUC), School of Pharmacy, Fudan University. The tumor, blood vessels, and lymph node UCL imaging was taken with a low‐power LED light (20 mW cm^−2^). For tumor imaging, the sarcoma 180‐bearing nude mice were intravenously injected with 0.2 mL PEG‐UCL‐QDs (3 mg mL^−1^), and UCL images were obtained by UCL in vivo imaging system after 6 h. The tumor and major organs (heart, liver, spleen, kidney, lung) were imaged by UCL in vivo imaging system after being resected. Blood vessels UCL images of nude mouse in vivo and in situ were acquired immediately after intravenous injection with 0.2 mL PEG‐UCL‐QDs (3 mg mL^−1^). In vivo lymphatic UCL imaging was performed 30 min after intradermal injection of PEG‐UCL‐QDs (50 µL, 3 mg mL^−1^) or PEG‐Tm‐UCNP (50 µL, 3 mg mL^−1^) into the paw of the nude mouse. The lymphatic drainage ex vivo UCL imaging was also measured after removal of the lymph node from the body.

## Conflict of Interest

The authors declare no conflict of interest.

## Supporting information

Supplementary
