# Supplementary material for: Near‐Infrared Upconversion Luminescence and Bioimaging In Vivo Based on Quantum Dots
Source: Adv Sci (Weinh). 2019 Jan 18;6(5):1801834. doi: 10.1002/advs.201801834 (PMC6402406; doi:10.1002/advs.201801834)
Supplement: Supplementary file 1 — Supplementary [file ADVS-6-1801834-s001.pdf]

Copyright WILEY-VCH Verlag GmbH & Co. KGaA, 69469 Weinheim, Germany, 2019.

ADVANCED SCIENCE
Open Access

Supporting Information

for *Adv. Sci.,* DOI: 10.1002/advs.201801834

Near-Infrared Upconversion Luminescence and Bioimaging In Vivo Based on Quantum Dots

Xiaochen Qiu, Xingjun Zhu, Xianlong Su, Ming Xu, Wei Yuan, Qingyun Liu, Meng Xue, Yawei Liu, Wei Feng, and Fuyou Li**

Copyright WILEY-VCH Verlag GmbH & Co. KGaA, 69469 Weinheim, Germany, 2018.

Supporting Information

Near-Infrared Upconversion Luminescence and Bioimaging in vivo Based on Quantum Dots

Xiaochen Qiu, Xingjun Zhu, Xianlong Su, Ming Xu, Wei Yuan, Qingyun Liu, Meng Xue, Yawei Liu, Wei Feng* and Fuyou Li*

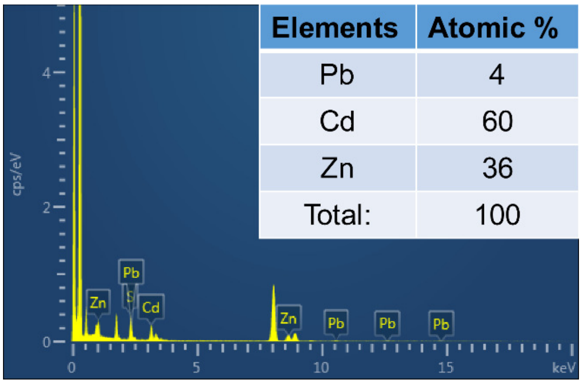

Figure S1. EDS spectrum of PbS/CdS/ZnS QDs.

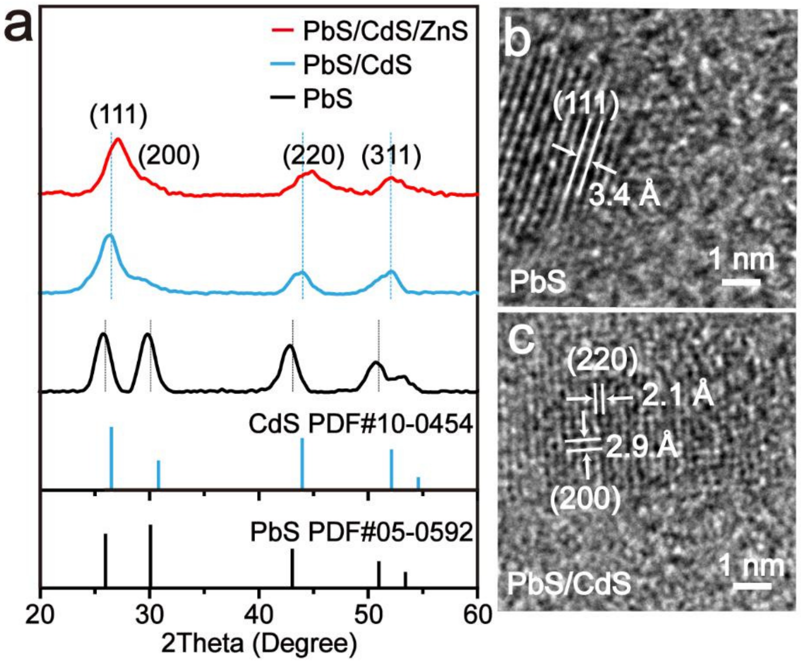

Figure S2. (a) XRD patterns of PbS, PbS/CdS, and PbS/CdS/ZnS QDs powders. Vertical bars (bottom) correspond to reference data of the rock-salt cubic crystalline structure of PbS (black) and zinc blende structure of CdS (blue) (PDF#05-0592 and PDF#10-0454, respectively). High-resolution TEM images of (b) the as-prepared PbS QDs and (c) PbS/CdS QDs.

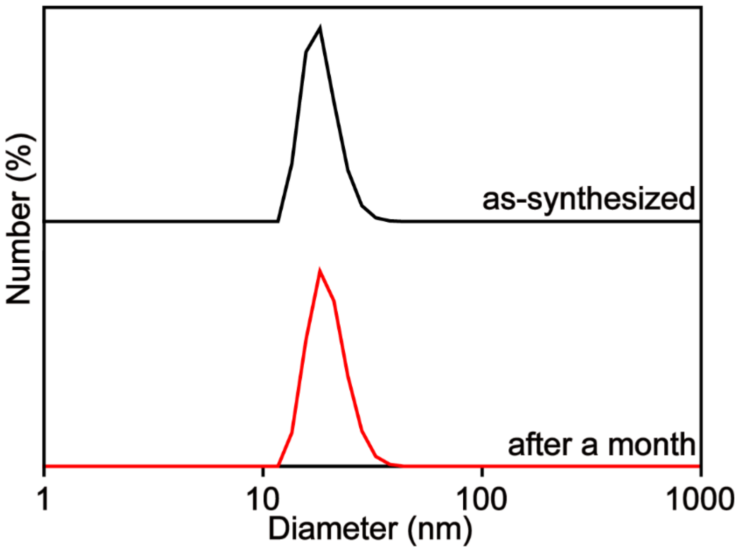

Figure 3. Dynamic light scattering patterns of PbS/CdS/ZnS-PEG in DI water as-synthesized and after storage for a month.

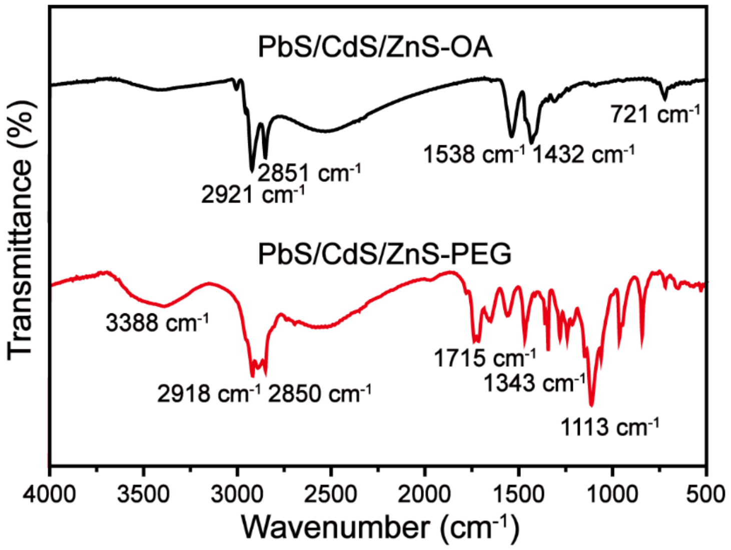

Figure S4. FT-IR spectra of the as-synthesized UCL-QDs-OA and UCL-QDs-PEG.

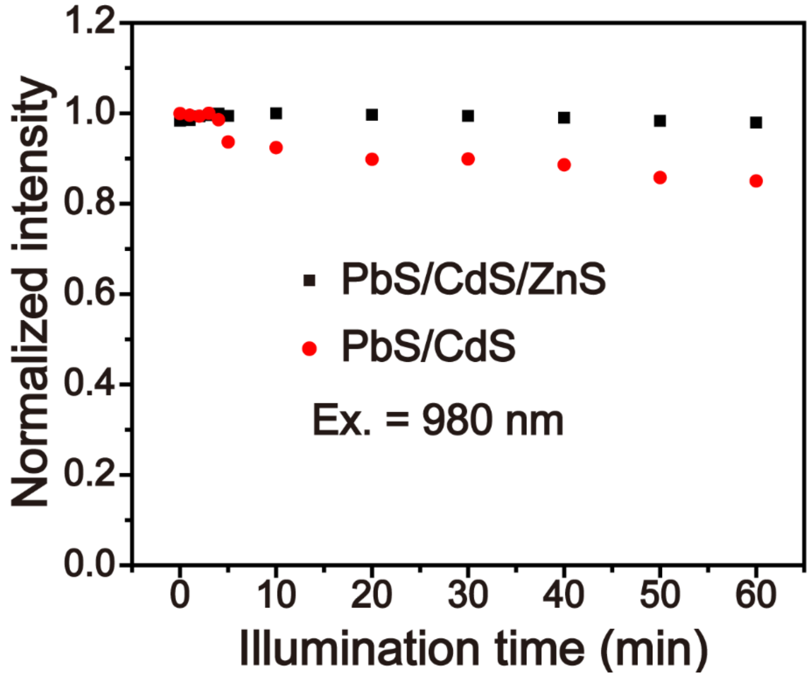

Figure S5. Time-dependent UCL intensity of PbS/CdS and PbS/CdS/Zn QDs aqueous solution excited under 980 nm, 200 mW/cm^2 laser.

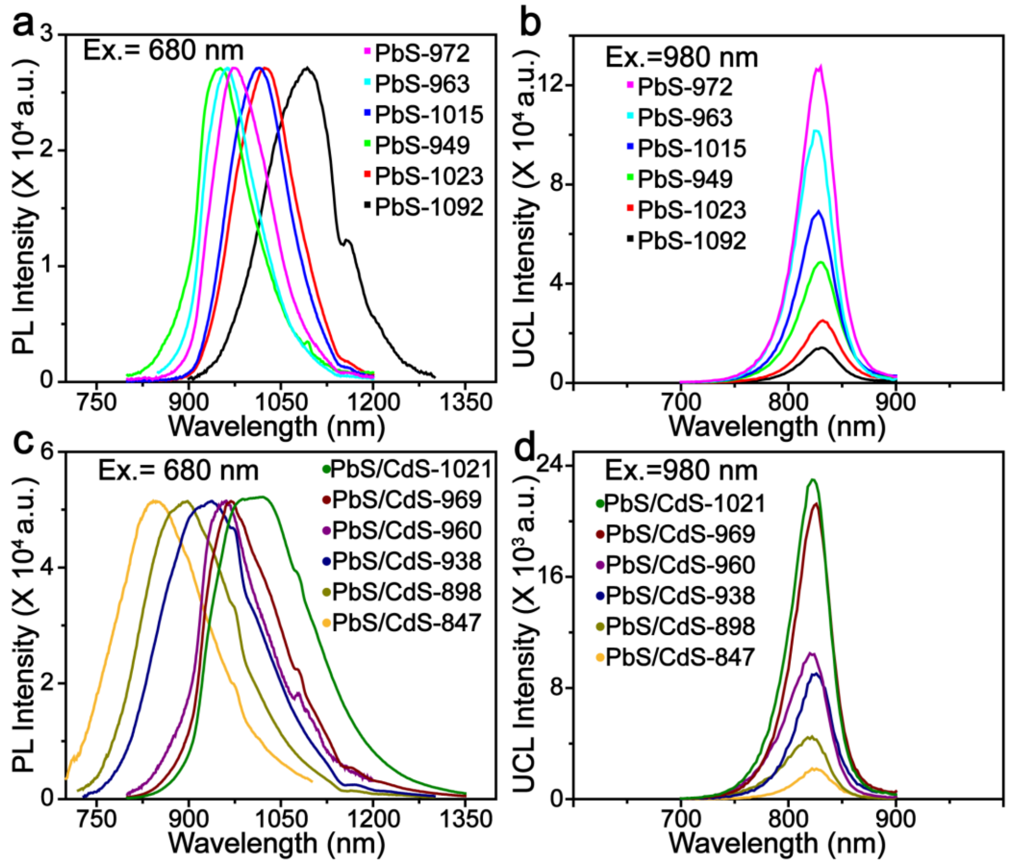

Figure S6. PL spectra (a and c) and corresponding UCL spectra (b and d).

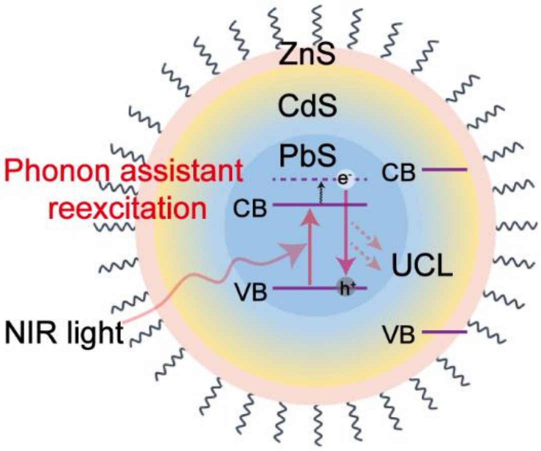

Figure S7. Schematic diagram of phonon-assisted single photon UCL of UCL-QDs.

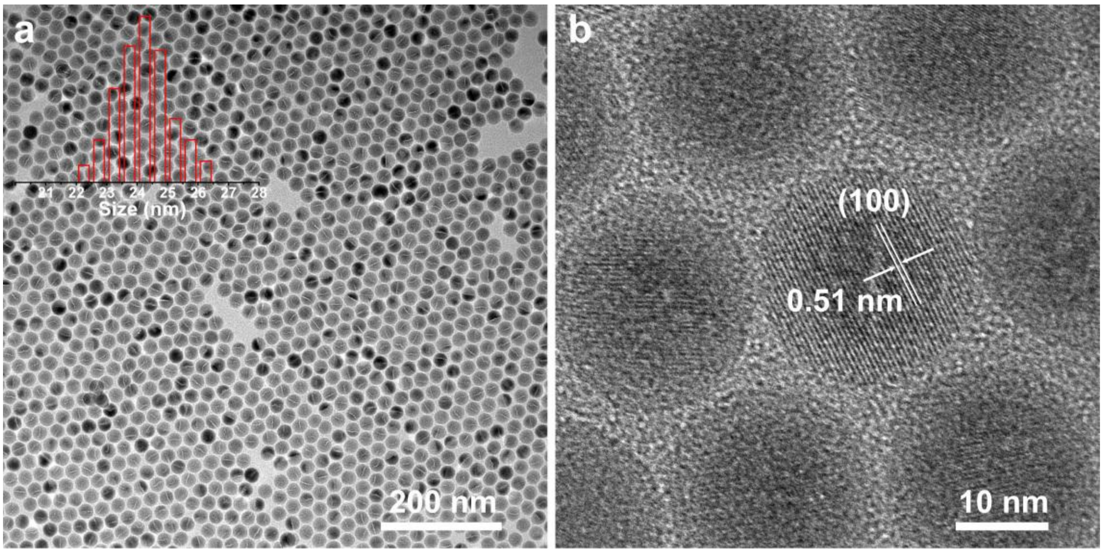

Figure S8. (a) TEM image and (b) HR-TEM image of $NaYbF_4$:0.5%Tm@$NaYF_4$ UCNPs. Inset of (a) shows the corresponding size distributions.

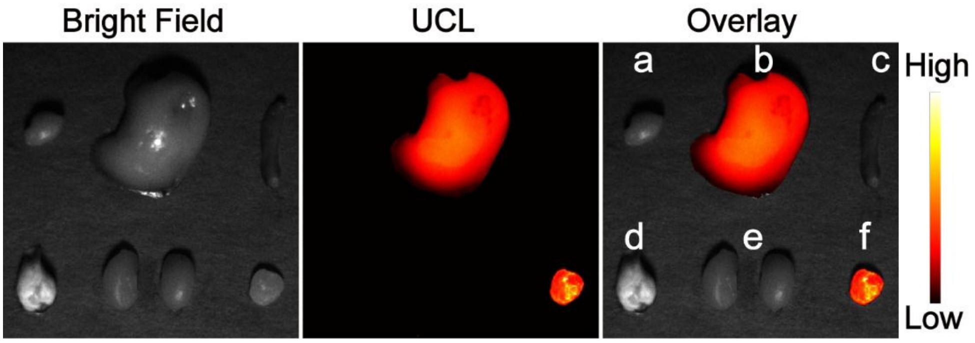

Figure S9. Ex vivo UCL imaging of tumor nude mice with intravenous injection of UCL-QDs (200 μL, 3 mg/mL) after 6 h. a: heart; b: liver; c: spleen; d: lung; e: kidney and f: tumor.

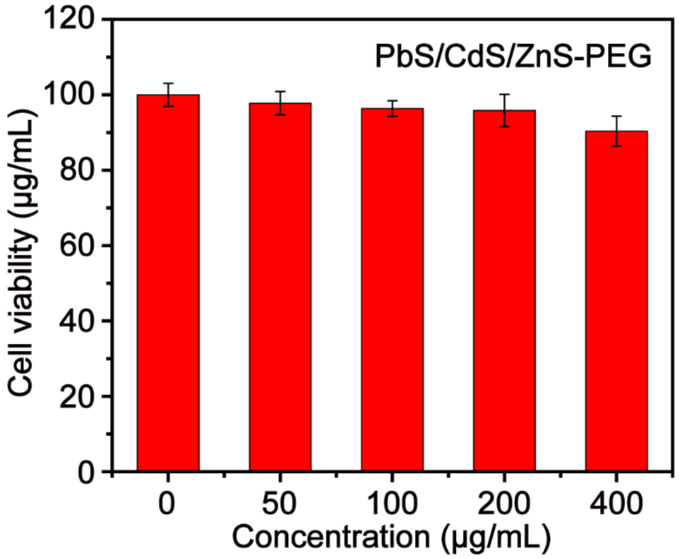

Figure S10. Cell viability values (%) estimated by the MTT proliferation test after incubation with different concentrations of UCL-QDs (0−400 μg/mL) for 24 h at 37 °C.
